# Racial and Ethnic Bias in Risk Prediction Models for Colorectal Cancer Recurrence When Race and Ethnicity Are Omitted as Predictors

**DOI:** 10.1001/jamanetworkopen.2023.18495

**Published:** 2023-06-15

**Authors:** Sara Khor, Eric C. Haupt, Erin E. Hahn, Lindsay Joe L. Lyons, Veena Shankaran, Aasthaa Bansal

**Affiliations:** 1 Comparative Health Outcomes, Policy, and Economics (CHOICE) Institute, University of Washington; 2 Department of Research and Evaluation, Kaiser Permanente Southern California, Pasadena; 3 Fred Hutchinson Cancer Center, Seattle, Washington; 4 Division of Medical Oncology, University of Washington School of Medicine, Seattle

## Abstract

**Importance:**

Including race and ethnicity as a predictor in clinical risk prediction algorithms has received increased scrutiny, but there continues to be a lack of empirical studies addressing whether simply omitting race and ethnicity from the algorithms will ultimately affect decision-making for patients of minoritized racial and ethnic groups.

**Objective:**

To examine whether including race and ethnicity as a predictor in a colorectal cancer recurrence risk algorithm is associated with racial bias, defined as racial and ethnic differences in model accuracy that could potentially lead to unequal treatment.

**Design, Setting, and Participants:**

This retrospective prognostic study was conducted using data from a large integrated health care system in Southern California for patients with colorectal cancer who received primary treatment between 2008 and 2013 and follow-up until December 31, 2018. Data were analyzed from January 2021 to June 2022.

**Main Outcomes and Measures:**

Four Cox proportional hazards regression prediction models were fitted to predict time from surveillance start to cancer recurrence: (1) a race-neutral model that explicitly excluded race and ethnicity as a predictor, (2) a race-sensitive model that included race and ethnicity, (3) a model with 2-way interactions between clinical predictors and race and ethnicity, and (4) separate models by race and ethnicity. Algorithmic fairness was assessed using model calibration, discriminative ability, false-positive and false-negative rates, positive predictive value (PPV), and negative predictive value (NPV).

**Results:**

The study cohort included 4230 patients (mean [SD] age, 65.3 [12.5] years; 2034 [48.1%] female; 490 [11.6%] Asian, Hawaiian, or Pacific Islander; 554 [13.1%] Black or African American; 937 [22.1%] Hispanic; and 2249 [53.1%] non-Hispanic White). The race-neutral model had worse calibration, NPV, and false-negative rates among racial and ethnic minority subgroups than non-Hispanic White individuals (eg, false-negative rate for Hispanic patients: 12.0% [95% CI, 6.0%-18.6%]; for non-Hispanic White patients: 3.1% [95% CI, 0.8%-6.2%]). Adding race and ethnicity as a predictor improved algorithmic fairness in calibration slope, discriminative ability, PPV, and false-negative rates (eg, false-negative rate for Hispanic patients: 9.2% [95% CI, 3.9%-14.9%]; for non-Hispanic White patients: 7.9% [95% CI, 4.3%-11.9%]). Inclusion of race interaction terms or using race-stratified models did not improve model fairness, likely due to small sample sizes in subgroups.

**Conclusions and Relevance:**

In this prognostic study of the racial bias in a cancer recurrence risk algorithm, removing race and ethnicity as a predictor worsened algorithmic fairness in multiple measures, which could lead to inappropriate care recommendations for patients who belong to minoritized racial and ethnic groups. Clinical algorithm development should include evaluation of fairness criteria to understand the potential consequences of removing race and ethnicity for health inequities.

## Key Points

**Question:**

Is omitting race and ethnicity as a predictor in colorectal cancer recurrence risk prediction models associated with racial and ethnic bias?

**Findings:**

In this prognostic study with 4230 patients with colorectal cancer, 4 prediction models for risk of postoperative cancer recurrence were developed and validated. Explicitly considering race and ethnicity as a predictor improved model predictive performance among racial and ethnic minority patients and increased algorithmic fairness in multiple performance measures.

**Meaning:**

These findings suggest that implementing clinical risk models that simply omit race and ethnicity may result in worse prediction accuracy for racial and ethnic minority groups that may lead to inappropriate care recommendations that ultimately contribute to health disparities.

## Teaser

This prognostic study examines whether including race and ethnicity as a predictor in a clinical risk model for colorectal cancer (CRC) recurrence is associated with racial bias, defined as differences in model accuracy that could lead to unequal treatment among minoritized racial and ethnic groups.

## Introduction

There is currently a lack of consensus on whether and how race and ethnicity should be included in clinical risk prediction models that are used to guide health care decisions.^1^ The inclusion of race and ethnicity in clinical decision-making has been highly scrutinized because of concerns over racial profiling and the subsequent unequal treatment that may be a result of judging an individual’s risks differentially based on differences in averages in the racial and ethnic groups to which they belong.^2^ Moreover, race and ethnicity is a social construct and its associations with health care utilization and outcomes reflect the associations of structural racism and inequalities with health.^3^ There are concerns that the use of race and ethnicity as a predictor may seem to reify race and ethnicity from social constructs to meaningful biological variables. As such, there have been calls to remove race and ethnicity in clinical algorithms or omit race and ethnicity when using existing risk calculators,^4,5,6^ and some institutions have formally removed race and ethnicity from existing clinical algorithms.^7,8^

On the other hand, in the clinical context, where accurate prognostication is the goal, developers and users of clinical risk prediction tools are concerned that excluding race and ethnicity could harm all groups by reducing predictive accuracy and especially disadvantage minority racial and ethnic groups in situations when prognostic differences exist across different racial and ethnic groups,^1,9^ likely due to structural inequalities. Recent studies have shown that omitting race in an estimated glomerular filtration rate (GFR) equation resulted in differential underestimation of filtration rates among Black vs White individuals, which could lead to systematic differences in appropriate care between groups and further exacerbate health disparities.^10,11,12^ Removing race from the GFR equation also created estimates that attenuate the racial differences in risk of kidney failure and mortality between Black and White patients, potentially misdirecting attention from understanding and addressing the racial disparities in kidney function and mortality risk.^10^ In lung and breast cancer screening, simulation studies have shown that clinical guidelines and algorithms that explicitly consider race and ethnicity instead of applying uniform screening criteria for all groups can potentially expand screening eligibility for higher-risk subgroups and reduce racial and ethnic disparities,^13,14^ underscoring the importance of considering race and ethnicity in algorithms that guide these decisions.

Currently, many clinical risk algorithms include race and ethnicity as a predictor.^15,16,17,18,19^ In colorectal cancer (CRC), multiple algorithms have been developed to predict patients’ recurrence risk after surgical resection and treatment of the primary tumor.^18,20,21,22,23^ The goal of these models is to tailor postoperative surveillance strategies for cancer survivors based on their risk of recurrence, thereby maximizing the benefits while minimizing the harm and burden of intensive surveillance tests and visits.^24,25,26,27^ One of the most recent studies^18^ found race to be a significantly associated with recurrence, and the authors included race as a factor in their risk calculator. Despite the increase in calls to reconsider the use of race and ethnicity in clinical prediction algorithms, it remains unclear whether the simple removal of race and ethnicity from algorithms such as this one will ultimately improve care decisions for patients of minoritized racial and ethnic groups.

In this study, we used a CRC recurrence risk prediction model as a case study to examine whether including or excluding race and ethnicity affected algorithmic bias. Specifically, we (1) examined racial bias, defined as differential model accuracy across racial and ethnic groups that could potentially lead to unequal treatment, and (2) assessed whether the inclusion of race and ethnicity as a predictor in the algorithm was associated with racial bias. Results from this study will provide empirical evidence to inform the debate around including vs excluding race and ethnicity in clinical algorithms and have important implications on the incorporation of race and ethnicity in CRC recurrence surveillance decisions to reduce potential disparities.

## Methods

### Data Source and Study Population

This study used linked cancer registry and electronic health record data from Kaiser Permanente Southern California (KPSC), a large integrated health care organization that provides insurance and comprehensive health care to more than 4.7 million members at 15 hospitals in Southern California. The membership of KPSC is socioeconomically diverse and reflects that of the Southern California census population.^28^ The linked data set contains information on membership, demographic characteristics, diagnoses, procedures, drugs, hospice use, and death.

Our study cohort included adult patients diagnosed with stage I to III CRC who had a resection between 2008 and 2013 (eFigure 1 in Supplement 1), with follow-up through December 31, 2018. Institutional review board approval was obtained from the University of Washington. A waiver of informed consent was granted by the review board because this research presents no more than minimal risk. This study followed the Transparent Reporting of a Multivariable Prediction Model for Individual Prognosis or Diagnosis (TRIPOD) reporting guideline for diagnostic and prognostic studies.

### Study Outcome

The outcome of interest was CRC recurrence. We identified recurrence using diagnosis codes and health care utilization patterns associated with metastatic disease deemed valid in previous studies and validated in our data (eAppendix 1 in Supplement 1).^29,30^

### Statistical Analysis

#### Model Development

We applied 4 prediction modeling strategies that differed in how they handled the race and ethnicity variable. The race-neutral model excluded race and ethnicity as a predictor. The race-sensitive model included race and ethnicity as a predictor as well as an additional interaction term between race and ethnicity and stage to allow for differential associations between race and ethnicity and cancer recurrence by stage of diagnosis, as seen in a previous study.^18^ The interaction model included interaction terms between race and ethnicity and all other covariates. Finally, the race-stratified model created a separate model for each racial and ethnic group that included all covariates, allowing each group to have its own set of coefficients.

All models used Cox proportional hazards regression with time from the start of surveillance to recurrence as the outcome and included covariates previously shown to be associated with cancer recurrence.^18^ Race and ethnicity information was obtained from membership or utilization data, preferred language, and birth certificates.^31^ Race and ethnicity categories included Asian, Hawaiian, or Pacific Islander; Black or African American; Hispanic; non-Hispanic White; and multiracial or other. There was no unknown or missing race and ethnicity. The multiracial or other subgroup was excluded from the analyses due to small sample size. eAppendix 2 in Supplement 1 shows model development details.

We used 5-fold cross validation in which we randomly split the entire data set into 5 equal parts, fitted the models to 4 parts of the data (training set), and obtained 3-year predicted risk of recurrence on the remaining part (test set) using the function predictionSurvProb (pec package in R software). This process was repeated 5 times to obtain predictions for all individuals. These predictions were then evaluated for algorithm fairness.

#### Statistical Criteria for Evaluating Algorithmic Fairness

We examined algorithmic fairness of each model across racial and ethnic groups using 4 sets of statistical criteria (eTable in Supplement 1). First, we assessed equal calibration within groups visually and using calibration intercept and slope. Equal discriminative ability was assessed using area under the receiver operating characteristic curve (AUC).^32^ Equal false-positive rates (FPRs) and false-negative rates (FNRs) were assessed at a 5% risk threshold, and equal positive predictive values (PPVs) and negative predictive values (NPVs) were also assessed at a 5% risk threshold.

There is no single accepted criterion for evaluating algorithmic fairness. Equal calibration within groups has been discussed as a necessary condition for algorithmic fairness^33^ and is often considered an important model property in medicine, as it captures accuracy over the entire spectrum of risk.^34^ In settings where care decisions are made using predefined or commonly accepted absolute risk thresholds, threshold-specific measures such as FPR, FNR, PPV, and NPV may also be clinically relevant.^35^ We used a 5% absolute risk threshold to evaluate these metrics, which approximately corresponded to the 25th percentile of risk prediction. This low-risk threshold was chosen for the CRC recurrence surveillance setting where the sensitivity of the algorithm is critical, ie, the harms of missing a recurrence far outweigh the harms of an unnecessary test. As a sensitivity analysis we varied the risk thresholds from 0% to 30% and calculated FPR, FNR, PPV, and NPV.

For each criterion, racial bias was measured using the range difference, a commonly used health disparity measure calculated by subtracting the measure in the worst-performing group from that in the best-performing group.^36^ Finally, to evaluate the potential clinical benefit of the models, we estimated the standardized net benefits by race and ethnicity at the 5% risk threshold.^37^

All confidence intervals were obtained using 1000 bootstrap samples. All analyses were performed using R version 4.0.4 (R Project for Statistical Computing).

## Results

The final cohort comprised 4230 patients who underwent a CRC resection between 2008 and 2013 (mean [SD] age, 65.3 [12.5] years; 2034 [48.1%] female). Among these patients, 490 (11.6%) identified as Asian, Hawaiian, or Pacific Islander; 554 (13.1%), Black or African American; 937 (22.1%), Hispanic; and 2249 (53.1%), non-Hispanic White. The median (range) follow-up time was 5.8 (0-12) years from surveillance start. Patient characteristics, overall and by racial and ethnic groups, are shown in Table 1.

**Table 1.** Baseline Characteristics of Patients Who Underwent Colorectal Cancer Resection, Overall and by Racial and Ethnic Subgroup

Characteristic	Patients, No. (%)				
	All (N = 4230)	Non-Hispanic White (n = 2249)	Black or African American (n = 554)	Hispanic (n = 937)	Asian, Hawaiian, or Pacific Islander (n = 490)
Age, y					
Mean (SD)	65.3 (12.5)	67.4 (12.4)	65.0 (11.7)	61.6 (12.7)	62.7 (11.5)
Median (range)	65.0 (18.0-90.0)	68.0 (18.0-90.0)	65.0 (30.0-90.0)	61.0 (24.0-90.0)	63.0 (30.0-90.0)
Sex					
Male	2196 (51.9)	1164 (51.8)	257 (46.4)	514 (54.9)	261 (53.3)
Female	2034 (48.1)	1085 (48.2)	297 (53.6)	423 (45.1)	229 (46.7)
Stage					
I	1775 (42.0)	923 (41.0)	215 (38.8)	402 (42.9)	235 (48.0)
II	1310 (31.0)	727 (32.3)	180 (32.5)	279 (29.8)	124 (25.3)
III	1145 (27.1)	599 (26.6)	159 (28.7)	256 (27.3)	131 (26.7)
Tumor histology					
Mucinous	347 (8.2)	189 (8.4)	44 (7.9)	87 (9.3)	27 (5.5)
Nonmucinous adenocarcinoma	3883 (91.8)	2060 (91.6)	510 (92.1)	850 (90.7)	463 (94.5)
Total lymph nodes examined and PNR					
0-12 Nodes	1432 (33.9)	734 (32.6)	171 (30.9)	338 (36.1)	189 (38.6)
>12 Nodes, PNR 0	1896 (44.8)	1037 (46.1)	259 (46.8)	402 (42.9)	198 (40.4)
>12 Nodes, PNR 0-0.06	197 (4.7)	110 (4.9)	30 (5.4)	35 (3.7)	22 (4.5)
>12 Nodes, PNR 0.06-0.13	271 (6.4)	152 (6.8)	32 (5.8)	62 (6.6)	25 (5.1)
>12 Nodes, PNR 0.13-0.25	209 (4.9)	110 (4.9)	26 (4.7)	46 (4.9)	27 (5.5)
>12 Nodes, PNR ≥0.25	225 (5.3)	106 (4.7)	36 (6.5)	54 (5.8)	29 (5.9)
Pathologic T-stage					
T0/T1/Tis	1164 (27.5)	583 (25.9)	139 (25.1)	262 (28.0)	180 (36.7)
T2	831 (19.6)	455 (20.2)	110 (19.9)	185 (19.7)	81 (16.5)
T3	1997 (47.2)	1080 (48.0)	270 (48.7)	437 (46.6)	210 (42.9)
T4	238 (5.6)	131 (5.8)	35 (6.3)	53 (5.7)	19 (3.9)
Tumor site					
Colon	3257 (77.0)	1746 (77.6)	479 (86.5)	681 (72.7)	351 (71.6)
Rectum	973 (23.0)	503 (22.4)	75 (13.5)	256 (27.3)	139 (28.4)
Adjuvant chemotherapy	1398 (33.0)	706 (31.4)	179 (32.3)	340 (36.3)	173 (35.3)
Perineural invasion					
No	2286 (54.0)	1180 (52.5)	300 (54.2)	537 (57.3)	269 (54.9)
Yes	97 (2.3)	54 (2.4)	9 (1.6)	25 (2.7)	9 (1.8)
Unknown	1847 (43.7)	1015 (45.1)	245 (44.2)	375 (40.0)	212 (43.3)

Abbreviation: PNR, positive node ratio.

### Overall Unadjusted Recurrence Rates by Racial and Ethnic Subgroups

The overall unadjusted 3-year cumulative incidence of CRC recurrence was 11.2%. It differed across racial and ethnic subgroups: 12.5% among Asian, Hawaiian, or Pacific Islander patients, 13.8% among Black or African American patients, 13.0% among Hispanic patients, and 9.5% among non-Hispanic White patients (Figure).

**Figure.**
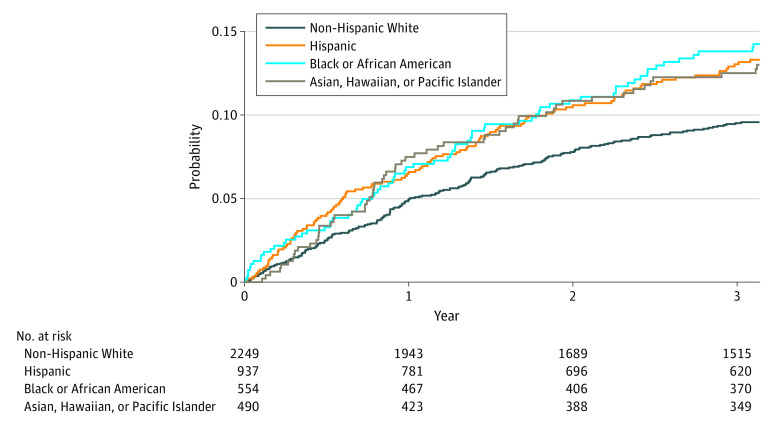
Cumulative Incidence of Colorectal Cancer Recurrence by Racial and Ethnic Groups

### Four Criteria of Algorithmic Fairness

#### Calibration

Calibration of the race-neutral model was excellent overall (calibration intercept, 0; [95% CI, −0.02 to 0.02]; slope, 1.00 [95% CI, 0.85 to 1.14]) but varied across racial and ethnic groups (eFigure 2A in Supplement 1). Specifically, calibration was best among non-Hispanic White patients, but worst among Black or African American patients, with a calibration slope 1.51 (95% CI, 1.08 to 1.93), suggesting that the model tended to overpredict recurrence risk for low-risk individuals and underpredict risk for high-risk individuals in this group. Adjusting for race and ethnicity (ie, the race-sensitive model) resulted in better calibration for Black or African American patients (slope, 1.09 [95% CI, 0.76 to 1.45]) (eFigure 2B in Supplement 1). The interaction model worsened the calibration accuracy in the overall cohort, with intercept significantly deviating from zero (0.02; 95% CI, 0.003 to 0.03) and slope significantly deviating from 1 (0.83; 95% CI, 0.71 to 0.97) (eFigure 2C in Supplement 1). Calibration among Asian, Hawaiian, or Pacific Islander patients was especially poor (slope, 0.58 [95% CI, 0.31 to 0.95]). The race-stratified model produced calibration results similar to the interaction model (eFigure 2D in Supplement 1). Overall, the race-sensitive model had good overall calibration and the most similar performance across groups, ie, the smallest difference in intercept and slope between the best- and worst-performing groups.

#### Discriminative Ability

Table 2 shows that the race-neutral model had good discrimination overall (AUC, 0.75 [95% CI, 0.72-0.77]), but it was variable across racial and ethnic groups: highest among Black or African American individuals (AUC, 0.79; 95% CI, 0.73-0.84) and lowest among Asian, Hawaiian, or Pacific Islander individuals (AUC, 0.72; 95% CI, 0.64-0.79). Explicitly including race and ethnicity as a predictor in the model (ie, the race-sensitive model) resulted in the same overall AUC but less variability across groups, with slightly lower AUC for the Black or African American group (AUC, 0.76; 95% CI, 0.70-0.82). Adding race and ethnicity interaction terms or stratifying by race resulted in lower AUCs for all groups compared with the race-neutral model, with greater reductions in AUCs for the racial and ethnic minority subgroups compared with non-Hispanic Whites. Overall, the race-sensitive model had the lowest racial bias, defined by the smallest range difference.

**Table 2.** Fairness Performance of Each Model Strategy in the Validation Data, Overall and by Racial and Ethnic Subgroups, and Racial Bias

Model	Model performance, % (95% CI)^a^					Racial bias, difference (95% CI), percentage point^a^^,^^b^
	All	Non-Hispanic White	Black or African American	Hispanic	Asian, Hawaiian, or Pacific Islander	
**Model discrimination, areas under the curve** ^c^						
Race-neutral	0.75 (0.72-0.77)	0.76 (0.72-0.79)	0.79 (0.73-0.84)	0.72 (0.66-0.77)	0.72 (0.64-0.79)	0.073 (0.030-0.17)
Race-sensitive	0.75 (0.72-0.77)	0.76 (0.72-0.79)	0.76 (0.7-0.82)	0.71 (0.66-0.77)	0.71 (0.64-0.78)	0.047 (0.024-0.15)
Interaction	0.73 (0.70-0.75)	0.75 (0.72-0.79)	0.75 (0.69-0.8)	0.68 (0.62-0.74)	0.68 (0.61-0.76)	0.070 (0.036-0.17)
Race-stratified	0.73 (0.70-0.76)	0.75 (0.72-0.78)	0.74 (0.68-0.8)	0.68 (0.62-0.75)	0.68 (0.61-0.76)	0.072 (0.038-0.17)
**False-positive rates at a prespecified 5% risk threshold**						
Race-neutral	68.2 (66.6-69.7)	69.1 (67.0-71.1)	65.0 (60.5-69.2)	70.0 (66.8-73.3)	64.4 (59.4-68.9)	5.6 (2.8-12)
Race-sensitive	69.9 (68.4-71.4)	64.1 (62-66.1)	74.9 (70.8-78.7)	75.7 (72.3-78.7)	81.9 (77.6-85.5)	17.8 (13.4-22.1)
Interaction	68.6 (67-70.1)	63.7 (61.6-65.8)	64.6 (59.8-68.8)	82.6 (79.9-85.2)	70.6 (66.2-74.8)	18.9 (16.1-23.6)
Race-stratified	68.7 (67-70.2)	61.6 (59.5-63.8)	69 (64.5-73.2)	85.7 (83.2-88.2)	70.6 (66.1-74.8)	24.1 (21.1-27.3)
**False-negative rates at a prespecified 5% risk threshold**						
Race-neutral	7.2 (4.7-9.8)	3.1 (0.8-6.2)	8.3 (2.1-15.5)	12.0 (6.0-18.6)	10.0 (2.3-18.8)	8.9 (4.6-17.4)
Race-sensitive	7.3 (4.9-9.9)	7.9 (4.3-11.9)	7 (1.8-13.4)	9.2 (3.9-14.9)	1.6 (−0.8-5.2)	7.6 (4.0-14.4)
Interaction	8.6 (6-11.2)	8.1 (4.2-12)	8.5 (2.7-15.2)	10.2 (4.3-16.3)	7.6 (1.3-15.1)	2.6 (1.8-12.8)
Race-stratified	8.5 (6-11.3)	8.4 (4.6-12.5)	8.3 (2.3-15.1)	8.3 (3.3-13.9)	9.5 (2.6-17.8)	1.2 (1.7-13.1)
**Positive predictive values at a prespecified 5% risk threshold**						
Race-neutral	15.1 (13.8-16.5)	13.0 (11.3-14.6)	20.1 (15.7-24.7)	16.9 (13.9-20.2)	16.3 (12.2-20.5)	7.1 (3.6-12.2)
Race-sensitive	14.8 (13.5-16.1)	13.3 (11.4-15.0)	18.1 (14.2-22.3)	16.3 (13.4-19.4)	14.4 (10.7-17.8)	4.9 (2.3-9.9)
Interaction	14.9 (13.5-16.1)	13.3 (11.5-15.0)	20.2 (15.9-24.7)	15.0 (12.2-17.9)	15.4 (11.5-19.3)	6.9 (3.3-11.9)
Race-stratified	14.9 (13.5-16.1)	13.6 (11.8-15.4)	19.2 (15.1-23.6)	14.8 (12.1-17.6)	15.2 (11.3-19.1)	5.5 (2.7-10.5)
**Negative predictive values at a prespecified 5% risk threshold**						
Race-neutral	96.9 (96.0-97.8)	98.8 (97.7-99.5)	95.6 (92.2-98.7)	93.6 (90.5-96.7)	96.0 (92.7-98.8)	5.1 (2.7-8.4)
Race-sensitive	96.8 (95.7-97.8)	97.6 (96.3-98.6)	95.4 (91.3-99.1)	93.9 (90.5-97.1)	98.7 (95.9-100)	4.8 (2.1-8.9)
Interaction	96.4 (95.3-97.4)	97.5 (96.3-98.5)	96.1 (92.8-99.2)	91.2 (86.1-95.9)	96.7 (93.2-100)	6.3 (2.2-12.2)
Race-stratified	96.4 (95.3-97.4)	97.5 (96.3-98.5)	95.6 (91.8-99.1)	91.2 (85.5-96.2)	95.8 (92.0-99.1)	6.3 (1.9-7.8)

^a^ The 95% CIs were obtained through 1000 bootstraps.

^b^ Racial bias is assessed using the range difference, computed by subtracting the measure in the worst-performing group from that in the best-performing group.

^c^ Area under the curve value of 0.5 suggests that the model performs no better than chance; 0.7 to 0.8 is considered acceptable, greater than 0.8 is considered excellent.

#### FPRs and FNRs

At a prespecified 5% risk threshold, the overall FPR and FNR were 68.2% (95% CI, 66.6%-69.7%) and 7.2% (95% CI, 4.7%-9.8%), respectively, in the race-neutral model. FPRs and FNRs differed across racial and ethnic groups, with Hispanic patients having the highest (ie, worst) FPR (70.0%; 95% CI, 66.8%-73.3%) and FNR (12.0%; 95% CI, 6.0%-18.6%) (Table 2). Non-Hispanic White patients had an FPR of 69.1% (95% CI, 67.0%-71.1%) and an FNR of 3.1% (95% CI, 0.8%-6.2%). Explicitly including race in the model (ie, the race-sensitive model) improved the FPRs and worsened the FNRs for the non-Hispanic White group (FPR: 64.1% [95% CI, 62.0%-66.1%]; FNR: 7.9% [95% CI, 4.3%-11.9%]) but worsened the FPRs and improved the FNRs for all other groups (eg, among Hispanic patients: FPR, 75.7% [95% CI, 72.3%-78.7%]; FNR, 9.2% [95% CI, 3.9%-14.9%]). Overall, the race-neutral model had the smallest difference in FPRs across groups, and the race-stratified model had the largest. Adding interaction terms and stratifying by race and ethnicity worsened overall FNRs slightly but resulted in more equal FNRs across racial and ethnic groups. Similar bias results were found when different risk thresholds were applied (eFigure 3 in Supplement 1).

#### PPV and NPV

At the 5% risk threshold, PPVs and NPVs varied across racial and ethnic groups in the race-neutral model (eg, non-Hispanic White: PPV, 13.0% [95% CI, 11.3%-14.6%]; NPV, 98.8% [95% CI, 97.7%-99.5%]; Black or African American: PPV, 20.1% [95% CI, 15.7%-24.7%]; NPV, 95.6% [95% CI, 92.2%-98.7%]) (Table 2) The race-sensitive, interaction, and stratified models all resulted in lower PPV than that in the race-neutral model, with the race-sensitive model having the lowest bias (4.9 percentage points; 95% CI, 2.3-9.9 percentage points). The race-sensitive model improved NPV for Asian, Hawaiian, or Pacific Islander individuals but had similar overall bias as the race-neutral model. Similar trends were found when different risk thresholds were applied (eFigure 4 in Supplement 1).

### Net Benefit

With a prespecified 5% risk threshold, we found that the race-sensitive model resulted in higher standardized net benefits among Asian, Hawaiian, or Pacific Islander individuals. However, the race-neutral model had higher net benefits among non-Hispanic White and Black or African American individuals (Table 3).

**Table 3.** Standardized Net Benefit of Each Model Strategy in the Validation Data, Overall and by Racial and Ethnic Subgroups at a 5% Risk Threshold^a^

Model	Net benefit, %				
	All	Non-Hispanic White	Black or African American	Hispanic	Asian, Hawaiian, or Pacific Islander
Race-neutral	65.4	62.7	72.5	65.2	65.7
Race-sensitive	64.6	60.4	70.9	66.2	67.5
Interaction	63.9	60.4	72.4	63.0	65.8
Race-stratified	63.9	61.1	71.4	63.8	63.8
Treat all	59.8	50.5	70.5	67.5	62.3

^a^ The standardized net benefit measures how much utility the model achieved relative to the maximum possible achievable utility.

## Discussion

This is the first study to our knowledge to examine racial bias in prediction algorithms for CRC recurrence for guiding postoperative cancer surveillance and to investigate the outcomes of omitting race and ethnicity as a predictor in these algorithms. Using rich electronic health records data from a large health system, we evaluated the algorithmic fairness in 4 risk prediction modeling approaches that varied in how race and ethnicity was considered. We demonstrated that racial bias existed even when race and ethnicity was excluded as a predictor, with worse calibration, NPV, and FNRs among racial and ethnic minority patients compared with non-Hispanic White patients. Furthermore, explicitly including race and ethnicity as a variable improved algorithmic fairness in calibration, discriminative ability, PPV and NPV, and FNR. Finally, we did not observe any additional benefit in fairness with the inclusion of race and ethnicity interaction terms or using race and ethnicity–stratified models, likely due to the smaller sample sizes in the subgroups.

Our study has important implications for developing clinical algorithms that are both accurate and fair. Although many clinicians, institutions, and lawmakers have called for the removal of race in clinical algorithms or the omission of race when using existing risk calculators,^4,5,6,7,8^ we demonstrated in the CRC recurrence setting that the simple removal of the race and ethnicity variable as a predictor could lead to higher racial bias in model accuracy and more inaccurate estimation of risk for racial and ethnic minority groups, which could potentially induce disparities by recommending inadequate or inappropriate surveillance and follow-up care disproportionately more often to patients of minoritized racial and ethnic groups.

The debate around whether race and ethnicity should be included in prediction models is related to the trade-off between 2 separate notions of fairness: fairness in treatment vs fairness in outcome.^38^ The former posits that all individuals with similar clinical characteristics should be offered the same care regardless of their race and ethnicity. The latter supports the idea that the more disadvantaged group should be given more resources to achieve similar health outcomes as the advantaged group. Both are important notions of fairness, but the former would recommend omitting race and ethnicity in clinical decision formulas while the latter would support the inclusion of race and ethnicity if racial and ethnic disparities in outcomes exist and if the effects of systemic racism could not be replaced with other clinical variables. There have been instances where researchers were able to identify other factors to replace race and ethnicity in clinical models without sacrificing subgroup model accuracy, achieving both notions of fairness.^10,11,39^ There is no one-size-fits all solution to designing equitable algorithms. What is considered fair and appropriate will likely depend on the decision context and long-term race-specific health and economic consequences of the proposed algorithmic interventions.^38^

Our findings resonate with some recent studies that also found evidence of differential model performance across racial and ethnic subgroups in other clinical risk algorithms,^40,41,42^ underscoring the importance of proactive and standardized examination of subgroup performance using appropriate fairness criteria before deploying a clinical risk prediction model for clinical decision-making, regardless of whether race and ethnicity are included as a predictor.^43^ It is important to note that measures for racial bias should be considered alongside overall performance when evaluating models to avoid selecting models that perform equally poorly for all groups.^44^ Assessing downstream health disparities of the different modeling strategies is also important. The current net benefits calculations assumed that the predefined risk threshold reflects that of the utilities associated with the benefits, harms, and costs of potential interventions for all subgroups. More complex decision analyses with complete information on subgroup-specific benefits, harms, and costs of the interventions are needed to assess the models’ long-term health disparity outcomes.

Finally, including race and ethnicity in the risk prediction model acknowledges that recurrence risk differs across racial and ethnic groups because of systemic racism but does not fix the racial disparity in recurrence. Instead, it represents a practical but short-term solution to ensure that the prediction model does not further contribute to the disparities by directing appropriate and timely care for all groups according to their needs. While this is important, it should not replace efforts to address the root causes of racial disparities of CRC outcomes—structural racism, social determinants of health, and differential access to health care and treatment.^45,46,47^

### Limitations

Our study has several limitations. First, our cohort consisted of insured patients from one integrated health system in Southern California, limiting the generalizability of the results. Second, the sample sizes of some racial and ethnic subgroups were small, limiting our ability to detect the benefits of including interaction terms or fitting race-stratified models. Future work should study the impact of such models on fairness using much larger racial and ethnic subgroups to allow for more flexible modeling. Third, as is common practice, we relied on a validated algorithm to identify recurrence outcomes using health care utilization patterns,^29,30^ which may have errors that can influence the racial algorithmic bias on the models, although the extent and direction of the effect remain unknown. Fourth, race and ethnicity are social constructs with many heterogeneous subgroups.^48^ Evaluating model performances using broad categories of race and ethnicity may hide the disparities in smaller distinct subpopulations. Fifth, our models were based on a previously published model for cancer recurrence. Models that include other potential predictors, such as comorbidities, biomarkers, or more recent treatment options, may alter the associations found in this study and should be evaluated for racial bias.

## Conclusions

Using a CRC recurrence risk prediction model, we found empirically that explicitly including race and ethnicity as a variable improved algorithmic fairness in multiple measures, including calibration, discriminative ability, PPV and NPV, and FNR. This has important fairness implications for the clinical community interested in algorithms that are both accurate and fair. Specifically, simply omitting race and ethnicity may result in worse prediction accuracy in subgroups that may lead to inappropriate care recommendations that ultimately perpetuate or contribute to disparities in health outcomes. Our results highlight the need to use clinically relevant fairness criteria to evaluate existing algorithms and understand the potential implications of removing the race and ethnicity variable on racial disparities in health outcomes.

## Notes

Supplement 1. **eFigure 1.** Inclusion and Exclusion Criteria **eAppendix 1.** Approach to Ascertaining the Model Outcome **eAppendix 2.** Model Development Details **eTable.** Statistical Criteria for Algorithmic Fairness **eFigure 2.** Comparison of Calibration Across Racial and Ethnic Groups in Each Model **eFigure 3.** False-Positive Rates (FPR) and False-Negative Rates (FNR) at Different Risk Thresholds, by Model Type and Race and Ethnicity **eFigure 4.** Positive Predictive Value (PPV) and Negative Predictive Value (NPV) at Different Risk Thresholds, by Model Type and Race and Ethnicity
eReferences.

Supplement 2.
Data Sharing Statement

## References

[1] Paulus JK, Kent DM. Race and ethnicity: a part of the equation for personalized clinical decision making? Circ Cardiovasc Qual Outcomes. 2017;10(7):e003823. doi:10.1161/CIRCOUTCOMES.117.003823 28687570 PMC5558842

[2] Paulus JK, Kent DM. Predictably unequal: understanding and addressing concerns that algorithmic clinical prediction may increase health disparities. NPJ Digit Med. 2020;3:99. doi:10.1038/s41746-020-0304-9 32821854 PMC7393367

[3] Bailey ZD, Krieger N, Agénor M, Graves J, Linos N, Bassett MT. Structural racism and health inequities in the USA: evidence and interventions. Lancet. 2017;389(10077):1453-1463. doi:10.1016/S0140-6736(17)30569-X 28402827

[4] Cerdeña JP, Plaisime MV, Tsai J. From race-based to race-conscious medicine: how anti-racist uprisings call us to act. Lancet. 2020;396(10257):1125-1128. doi:10.1016/S0140-6736(20)32076-6 33038972 PMC7544456

[5] Vyas DA, Eisenstein LG, Jones DS. Hidden in plain sight—reconsidering the use of race correction in clinical algorithms. N Engl J Med. 2020;383(9):874-882. doi:10.1056/NEJMms2004740 32853499

[6] House Committee on Ways and Means. Fact versus fiction: clinical decision support tools and the mis(use) of race. Accessed May 15, 2023. https://democrats-waysandmeans.house.gov/sites/democrats.waysandmeans.house.gov/files/documents/Fact%20Versus%20Fiction%20Clinical%20Decision%20Support%20Tools%20and%20the%20%28Mis%29Use%20of%20Race%20%282%29.pdf

[7] UC Davis Health. UC Davis drops race-based reference ranges from a standard kidney test. May 18, 2021. Accessed May 15, 2023. https://health.ucdavis.edu/news/headlines/uc-davis-drops-race-based-reference-ranges-from-a-standard-kidney-test/2021/05

[8] Nkinsi NT, Young BA. How the University of Washington implemented a change in eGFR reporting. Kidney360. 2022;3(3):557-560. doi:10.34067/KID.0006522021 35582183 PMC9034824

[9] Manski CF. Patient-centered appraisal of race-free clinical risk assessment. Health Econ. Published online July 5, 2022. doi:10.1002/hec.4569 35791466

[10] Gutiérrez OM, Sang Y, Grams ME, ; Chronic Kidney Disease Prognosis Consortium. Association of estimated GFR calculated using race-free equations with kidney failure and mortality by Black vs non-Black race. JAMA. 2022;327(23):2306-2316. doi:10.1001/jama.2022.8801 35667006 PMC9171658

[11] Inker LA, Eneanya ND, Coresh J, ; Chronic Kidney Disease Epidemiology Collaboration. New creatinine- and cystatin C-based equations to estimate GFR without race. N Engl J Med. 2021;385(19):1737-1749. doi:10.1056/NEJMoa2102953 34554658 PMC8822996

[12] Diao JA, Wu GJ, Taylor HA, . Clinical implications of removing race from estimates of kidney function. JAMA. 2021;325(2):184-186. doi:10.1001/jama.2020.22124 33263721 PMC7711563

[13] Han SS, Chow E, Ten Haaf K, . Disparities of national lung cancer screening guidelines in the US population. J Natl Cancer Inst. 2020;112(11):1136-1142. doi:10.1093/jnci/djaa013 32040195 PMC7669226

[14] Chapman CH, Schechter CB, Cadham CJ, . Identifying equitable screening mammography strategies for Black women in the United States using simulation modeling. Ann Intern Med. 2021;174(12):1637-1646. doi:10.7326/M20-6506 34662151 PMC9997651

[15] Breast Cancer Surveillance Consortium. Breast Cancer Surveillance Consortium risk calculator. Accessed May 15, 2023. https://tools.bcsc-scc.org/BC5yearRisk/intro.htm

[16] MD Anderson Cancer Center. Rectal cancer survival calculator. Accessed May 15, 2023. http://www3.mdanderson.org/app/medcalc/index.cfm?pagename=rectumcancer

[17] Eneanya ND, Yang W, Reese PP. Reconsidering the consequences of using race to estimate kidney function. JAMA. 2019;322(2):113-114. doi:10.1001/jama.2019.5774 31169890

[18] Zafar SN, Hu CY, Snyder RA, . Predicting risk of recurrence after colorectal cancer surgery in the United States: an analysis of a special Commission on Cancer national study. Ann Surg Oncol. 2020;27(8):2740-2749. doi:10.1245/s10434-020-08238-7 32080809

[19] Shahian DM, Jacobs JP, Badhwar V, . The Society of Thoracic Surgeons 2018 adult cardiac surgery risk models: part 1—background, design considerations, and model development. Ann Thorac Surg. 2018;105(5):1411-1418. doi:10.1016/j.athoracsur.2018.03.002 29577925

[20] Ho D, Tan IBH, Motani M. Predictive models for colorectal cancer recurrence using multi-modal healthcare data. *CHIL 21: Proc Conference Health Inference Learning*. 2021:204-213. doi:10.1145/3450439.3451868

[21] Peng J, Ding Y, Tu S, . Prognostic nomograms for predicting survival and distant metastases in locally advanced rectal cancers. PLoS One. 2014;9(8):e106344. doi:10.1371/journal.pone.0106344 25171093 PMC4149564

[22] Honda M, Oba K, Akiyoshi T, . Development and validation of a prognostic nomogram for colorectal cancer after radical resection based on individual patient data from three large-scale phase III trials. Oncotarget. 2017;8(58):99150-99160. doi:10.18632/oncotarget.21845 29228760 PMC5716800

[23] Valentini V, van Stiphout RG, Lammering G, . Nomograms for predicting local recurrence, distant metastases, and overall survival for patients with locally advanced rectal cancer on the basis of European randomized clinical trials. J Clin Oncol. 2011;29(23):3163-3172. doi:10.1200/JCO.2010.33.1595 21747092

[24] Makhoul R, Alva S, Wilkins KB. Surveillance and survivorship after treatment for colon cancer. Clin Colon Rectal Surg. 2015;28(4):262-270. doi:10.1055/s-0035-1564435 26648797 PMC4655110

[25] Hafslund B, Espehaug B, Nortvedt MW. Effects of false-positive results in a breast screening program on anxiety, depression and health-related quality of life. Cancer Nurs. 2012;35(5):E26-E34. doi:10.1097/NCC.0b013e3182341ddb 22067696

[26] Søreide K. Endoscopic surveillance after curative surgery for sporadic colorectal cancer: patient-tailored, tumor-targeted or biology-driven? Scand J Gastroenterol. 2010;45(10):1255-1261. doi:10.3109/00365521.2010.496492 20553114

[27] Augestad KM, Rose J, Crawshaw B, Cooper G, Delaney C. Do the benefits outweigh the side effects of colorectal cancer surveillance? a systematic review. World J Gastrointest Oncol. 2014;6(5):104-111. doi:10.4251/wjgo.v6.i5.104 24834140 PMC4021326

[28] Koebnick C, Langer-Gould AM, Gould MK, . Sociodemographic characteristics of members of a large, integrated health care system: comparison with US Census Bureau data. Perm J. 2012;16(3):37-41. doi:10.7812/TPP/12-031 23012597 PMC3442759

[29] Hassett MJ, Uno H, Cronin AM, Carroll NM, Hornbrook MC, Ritzwoller D. Detecting lung and colorectal cancer recurrence using structured clinical/administrative data to enable outcomes research and population health management. Med Care. 2017;55(12):e88-e98. doi:10.1097/MLR.0000000000000404 29135771 PMC4732933

[30] Hassett MJ, Ritzwoller DP, Taback N, . Validating billing/encounter codes as indicators of lung, colorectal, breast, and prostate cancer recurrence using 2 large contemporary cohorts. Med Care. 2014;52(10):e65-e73. doi:10.1097/MLR.0b013e318277eb6f 23222531 PMC3600389

[31] Derose SF, Contreras R, Coleman KJ, Koebnick C, Jacobsen SJ. Race and ethnicity data quality and imputation using US Census data in an integrated health system: the Kaiser Permanente Southern California experience. Med Care Res Rev. 2013;70(3):330-345. doi:10.1177/1077558712466293 23169896

[32] Hosmer DW, Lemeshow S. *Applied Logistic Regression*. Second edition. Wiley; 2000.

[33] Hedden B. On statistical criteria of algorithmic fairness. Philos Public Aff. 2021;49(2):209-231. doi:10.1111/papa.12189

[34] Alba AC, Agoritsas T, Walsh M, . Discrimination and calibration of clinical prediction models: users’ guides to the medical literature. JAMA. 2017;318(14):1377-1384. doi:10.1001/jama.2017.12126 29049590

[35] Rajkomar A, Hardt M, Howell MD, Corrado G, Chin MH. Ensuring fairness in machine learning to advance health equity. Ann Intern Med. 2018;169(12):866-872. doi:10.7326/M18-1990 30508424 PMC6594166

[36] Ahn J, Harper S, Yu M, Feuer EJ, Liu B, Luta G. Variance estimation and confidence intervals for 11 commonly used health disparity measures. JCO Clin Cancer Inform. 2018;2:1-19. doi:10.1200/CCI.18.00031 30652598 PMC6873904

[37] Vickers AJ, van Calster B, Steyerberg EW. A simple, step-by-step guide to interpreting decision curve analysis. Diagn Progn Res. 2019;3:18. doi:10.1186/s41512-019-0064-7 31592444 PMC6777022

[38] Corbett-Davis S, Goel S. The measure and mismeasure of fairness: a critical review of fair machine learning. arXiv. Preprint posted online July 31, 2018. doi:10.48550/arXiv.1808.00023

[39] Shaikh N, Lee MC, Stokes LR, . Reassessment of the role of race in calculating the risk for urinary tract infection: a systematic review and meta-analysis. JAMA Pediatr. 2022;176(6):569-575. doi:10.1001/jamapediatrics.2022.0700 35435935 PMC9016605

[40] Obermeyer Z, Powers B, Vogeli C, Mullainathan S. Dissecting racial bias in an algorithm used to manage the health of populations. Science. 2019;366(6464):447-453. doi:10.1126/science.aax2342 31649194

[41] Coley RY, Johnson E, Simon GE, Cruz M, Shortreed SM. Racial/ethnic disparities in the performance of prediction models for death by suicide after mental health visits. JAMA Psychiatry. 2021;78(7):726-734. doi:10.1001/jamapsychiatry.2021.0493 33909019 PMC8082428

[42] Huang J, Galal G, Etemadi M, Vaidyanathan M. Evaluation and mitigation of racial bias in clinical machine learning models: scoping review. JMIR Med Inform. 2022;10(5):e36388. doi:10.2196/36388 35639450 PMC9198828

[43] Reyna MA, Nsoesie EO, Clifford GD. Rethinking algorithm performance metrics for artificial intelligence in diagnostic medicine. JAMA. 2022;328(4):329-330. doi:10.1001/jama.2022.10561 35802382 PMC11798426

[44] Brake DL. When equality leaves everyone worse off: the problem of leveling down in equality law. William Mary Law Rev. 2004;46(2):513-618. Accessed May 15, 2023. https://scholarship.law.wm.edu/cgi/viewcontent.cgi?article=1268&context=wmlr

[45] Clouston SAP, Acker J, Rubin MS, Chae DH, Link BG. Fundamental social causes of inequalities in colorectal cancer mortality: a study of behavioral and medical mechanisms. Heliyon. 2020;6(3):e03484. doi:10.1016/j.heliyon.2020.e03484 32190753 PMC7068626

[46] Thornton RL, Glover CM, Cené CW, Glik DC, Henderson JA, Williams DR. Evaluating strategies for reducing health disparities by addressing the social determinants of health. Health Aff (Millwood). 2016;35(8):1416-1423. doi:10.1377/hlthaff.2015.1357 27503966 PMC5524193

[47] Davis RE, Trickey AW, Abrahamse P, Kato I, Ward K, Morris AM. Association of cumulative social risk and social support with receipt of chemotherapy among patients with advanced colorectal cancer. JAMA Netw Open. 2021;4(6):e2113533. doi:10.1001/jamanetworkopen.2021.13533 34106262 PMC8190628

[48] Kauh TJ, Read JG, Scheitler AJ. The critical role of racial/ethnic data disaggregation for health equity. Popul Res Policy Rev. 2021;40(1):1-7. doi:10.1007/s11113-020-09631-6 33437108 PMC7791160

