# Supplementary material for: Racial and Ethnic Bias in Risk Prediction Models for Colorectal Cancer Recurrence When Race and Ethnicity Are Omitted as Predictors
Source: JAMA Netw Open. 2023 Jun 15;6(6):e2318495. doi: 10.1001/jamanetworkopen.2023.18495 (PMC10273018; doi:10.1001/jamanetworkopen.2023.18495)
Supplement: Supplement 1. — eFigure 1. Inclusion and Exclusion Criteria eAppendix 1. Approach to Ascertaining the Model Outcome eAppendix 2. Model Development Details eTable. Statistical Criteria for Algorithmic Fairness eFigure 2. Comparison of Calibration Across Racial and Ethnic Groups in Each Model eFigure 3. False-Positive Rates (FPR) and False-Negative Rates (FNR) at Different Risk Thresholds, by Model Type and Race and Ethnicity eFigure 4. Positive Predictive Value (PPV) and Negative Predictive Value (NPV) at Different Risk Thresholds, by Model Type and Race and Ethnicity eReferences. [file jamanetwopen-e2318495-s001.pdf]

Supplemental Online Content

Khor S, Haupt EC, Hahn EE, Lyons LJL, Shankaran V, Bansal A. Racial and ethnic bias in risk prediction models for colorectal cancer recurrence when race and ethnicity are omitted as predictors. *JAMA Netw Open.* 2023;6(6):e2318495. doi:10.1001/jamanetworkopen.2023.18495

eFigure 1. Inclusion and Exclusion Criteria

eAppendix 1. Approach to Ascertaining the Model Outcome

eAppendix 2. Model Development Details

eTable. Statistical Criteria for Algorithmic Fairness

eFigure 2. Comparison of Calibration Across Racial and Ethnic Groups in Each Model

eFigure 3. False-Positive Rates (FPR) and False-Negative Rates (FNR) at Different Risk Thresholds, by Model Type and Race and Ethnicity

eFigure 4. Positive Predictive Value (PPV) and Negative Predictive Value (NPV) at Different Risk Thresholds, by Model Type and Race and Ethnicity

eReferences.

This supplemental material has been provided by the authors to give readers additional information about their work.

© 2023 Khor S et al. *JAMA Network Open.*

eFigure 1. Inclusion and Exclusion Criteria

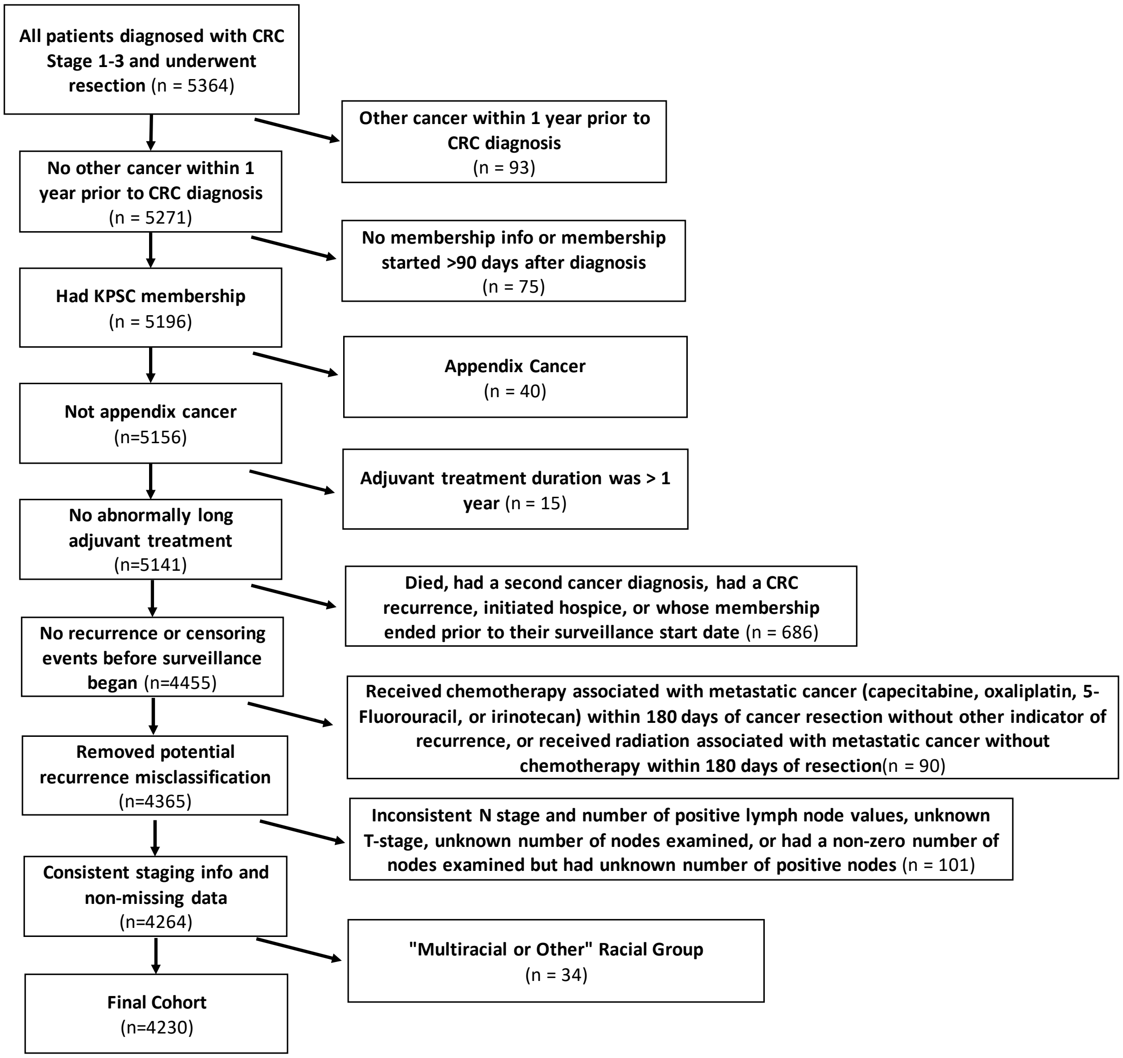

Description: We excluded patients diagnosed with appendix cancer, had a previous cancer diagnosis within one year prior to their CRC diagnosis, had no KPSC membership within 90 days of CRC diagnosis, or whose adjuvant treatment duration was unusually long (i.e., > 1 year). Cancer surveillance start was defined as 90 days after the end of primary surgery or adjuvant treatment. We excluded patients who died, had a second cancer diagnosis, had a CRC recurrence, initiated hospice, or whose membership ended prior to their surveillance start date. To avoid the misclassification of CRC recurrence, we further excluded patients who received chemotherapy associated with metastatic cancer (i.e., capecitabine, oxaliplatin, 5-Fluorouracil, or irinotecan) within 180 days of their cancer resection but had no other indicator of recurrence, and those who received radiation associated with metastatic cancer but without any chemotherapy within 180 days of their cancer resection. We also excluded those with inconsistent N stage and number of positive lymph node values, unknown T-stage, unknown number of nodes examined, or had a non-zero number of nodes examined but had unknown number of positive nodes. Finally, we excluded individuals in the “multiracial or other” racial/ethnic group due to small sample size.

Handling of Missing data: There was no missing outcome status as we relied on a validated algorithm to identify recurrence outcomes using healthcare utilization patterns (see eTable1). Patients with missing predictor information (T-stage, number of nodes examined, or had a non-zero number of nodes examined but had unknown number of positive nodes) were excluded as shown in diagram above. Unknown Perineural Invasion status was captured using an indicator variable.

© 2023 Khor S et al. *JAMA Network Open.*

eAppendix 1. Approach to Ascertaining the Model Outcome

Patients were considered having a recurrence if they had any of the following:

1) A prescription for any of the following adjuvant CRC drugs (fluorouracil, oxaliplatin, capecitabine) more than 90 days after the end of adjuvant therapy;
2) A prescription for any of the metastatic CRC drugs (irinotecan, cetixumab, panitumumab, bevacizumab, aflibercept, ziv-aflibercept, regorafenib, trifluridine, ramcirumab, nivolumab, pembrolizumab) anytime;
3) A prescription for any anti-cancer therapy associated with a metastatic ICD diagnosis code (ICD9: 197, 198, ICD10: C78, C79) anytime;
4) Received radiation therapy more than 90 days after the end of adjuvant therapy;
5) A primary CRC surgery procedure ≥ 225 days (7.5months) after KPSC Cancer Registry surgery date;
6) A metastatic surgery procedure;
7) Any imaging performed associated with a metastatic diagnosis, defined by having any imaging impression text in the exam summary from the radiologist that mentioned potential recurrence or evidence of metastatic disease and at least one occurrence of a metastatic cancer diagnosis code (ICD9: 197, 198, ICD10: C78, C79) within 30 days of the imaging date in the patients' history or encounter records; or
8) A hospice referral with a metastatic ICD diagnosis code.

A detailed chart review was performed in a random sample of 315 individuals to validate the recurrence outcome captured using this algorithm. Overall accuracy of the utilization-based recurrence outcome was high (positive predicted value 90%; negative predicted value 97%) and comparable to that found in other studies.[1,2]

© 2023 Khor S et al. *JAMA Network Open.*

eAppendix 2. Model Development Details

We applied four prediction modeling strategies that differed in how they handled the race/ethnicity variable. All models used Cox proportional hazards regression with time from the start of surveillance to recurrence as the outcome, with KPSC membership end, hospice initiation, second non-CRC primary cancer diagnosis, and end of study before recurrence treated as censoring events. Death before recurrence, a competing event, was infrequent (10%). We compared the risk estimates from the Cox model to those obtained using a competing risk regression (Fine and Gray) and saw minimal impact on estimates due to the relatively small proportion of patients who died before recurrence. Death was therefore treated as a censored observation to simplify the analysis.

For all models, we included variables previously shown to be predictive of cancer recurrence in the models.[3] The variables included were age, sex (male, female), cancer stage (AJCC v7), tumor histology, number of lymph nodes examined, positive node ratio (PNR), pathologic T-stage, tumor site (colon vs. rectum), adjuvant chemotherapy received, perineural invasion, and the interaction terms stage*adjuvant chemotherapy and stage*age. All covariates, except for adjuvant chemotherapy received, were measured at the time of diagnosis. All tumor information was obtained from the KPSC SEER-affiliated cancer registry. Tumor histology was defined using ICD-O-3 Histology codes: Non-mucinous adenocarcinoma (codes "8140", "8144", "8210", "8211", "8221","8255", "8260", "8261", "8262","8263", or "8574") and Mucinous neoplasms (codes “8480" and "8481"). The number of regional nodes found positive for cancer at pathological examination and the number of regional lymph nodes pathologically examined were obtained from the SEER Extent of Disease records. PNR was defined as the ratio of the number of positive lymph nodes to the total number of lymph nodes examined, which was calculated for patients with more than 12 nodes examined. Pathologic T-stage referred to T-stage per AJCC v6. Tumor site was identified using ICD-O-3 Site codes: colon (codes: C180, C182-189) and rectum (codes: C199, C209). The Collaborative Staging Site-Specific Factor 8 was used to identify perineural invasion status, which was dichotomize as Yes – Perineural invasion present vs. No – perineural invasion not present. All treatment information was extracted from pharmacy database and Electronic Medical Records. Receipt of adjuvant chemotherapy (Yes/No) was defined as the initiation of capecitabine, fluorouracil, or capecitabine within 90 days of surgery or radiation therapy (if received after surgery).

Race/ethnicity information was obtained from membership files, utilization data, preferred language, and birth certificates.[4] Self-reported race/ethnicity and official documents were given preference over other sources. Race/ethnicity categories included Non-Hispanic White, Hispanic, Black/African American, Asian/Hawaiian/Pacific Islander, and Multiracial or Other. There was no unknown or missing race/ethnicity. The “Multiracial or Other” subgroup was excluded from the analyses due to small sample size.

© 2023 Khor S et al. *JAMA Network Open.*

eTable. Statistical Criteria for Algorithmic Fairness

Description	How it was calculated
Equal Calibration within Groups[5,6]	
• For each possible predicted risk score, the proportion of patients experiencing a recurrence should be the same across racial/ethnic subgroups and equal to that risk score.<br>• Motivated by the idea that fairness requires a given risk score to have the same evidential value regardless of racial/ethnic group.[7]	For each racial/ethnic group, we plotted the observed Kaplan-Meier risks vs. the predicted recurrence risks across deciles of predicted risks. The predicted and expected risks were estimated using predictionSurvProb and calPlot (from the pec package). Calibration was assessed by the calibration intercept and slope. The intercept assesses calibration-in-the-large (or mean calibration),[8] with negative values suggesting overestimation and positive values suggesting underestimation. A slope <1 suggests that the estimated risks are too high for those with high risk and too low for patients at low risk. Slope >1 suggests that the risk estimates are too moderate.
Equal Discriminative Ability[9]	
• Motivated by the thought that a fair model should be able to correctly rank order individuals equally well between racial/ethnic groups.	Area under the receiver operating characteristic curve (AUC), which measures how well each model was at distinguishing between those with or without recurrence for each racial/ethnic group. Values range from 0 to 1. Value of 0.5 suggests that the model performs no better than chance; 0.7 to 0.8 is considered acceptable, > 0.8 is considered excellent.[10]
Equal False-Positive and False-Negative Rates[6,11,12]	
• Among those who truly are without a recurrence, the proportion falsely predicted to be positive (false-positive rate; FPR) should be the same across racial/ethnic groups.<br>• Similarly, among those who truly had a recurrence, the proportion falsely predicted to not have a recurrence (false-negative rate; FNR) should be the same across racial/ethnic groups.<br>• These two fairness criteria, sometimes referred to as "equalized odds", require that individuals from different groups with similar actual risk be treated the same by the algorithm.[11]	We evaluated the FNR and FPR at a 5% risk threshold, reflecting a hypothetical clinical scenario where intensive surveillance may be recommended for patients whose risks of recurrence within 3 years exceed 5%. Note that a lower risk cutoff (i.e. recommending more intensive surveillance for a larger proportion of patients) may be of interest for clinical scenarios where sensitivity of the algorithm is critical – the harms of missing a recurrence far outweigh the harms of an unnecessary test. A higher threshold, in contrast, weighs the relative harm of a false positive higher.
Equal Positive Predictive Value and Negative Predictive Value[6]	
• Among those who were predicted to have a recurrence (defined by risk above a pre-defined threshold), the proportion who actually experienced a recurrence (Positive Predictive Value; PPV) should be the same across racial/ethnic groups.<br>• Similarly, among those who were predicted to be recurrence-negative (defined by risk below or equal to a pre-defined threshold), the proportion who were actually recurrence-negative (negative predictive value, NPV) should be the same across racial/ethnic groups.<br>• These two criteria are similar to criterion 1 in that they are motivated by the idea that fairness requires a positive or negative prediction to have the same evidential value across all groups.	We evaluated the PPV and NPV at a 5% risk threshold.

© 2023 Khor S et al. *JAMA Network Open.*

eFigure 2. Comparison of Calibration Across Racial and Ethnic Groups in Each Model

The intercept assesses calibration-in-the-large (or mean calibration), with negative values suggesting overestimation and positive values suggesting underestimation. A slope <1 suggests that the estimated risks are too high for those with high risk and too low for patients at low risk. Slope >1 suggests that the risk estimates are too moderate. Values in brackets show the 95% confidence intervals obtained through 1000 bootstraps.

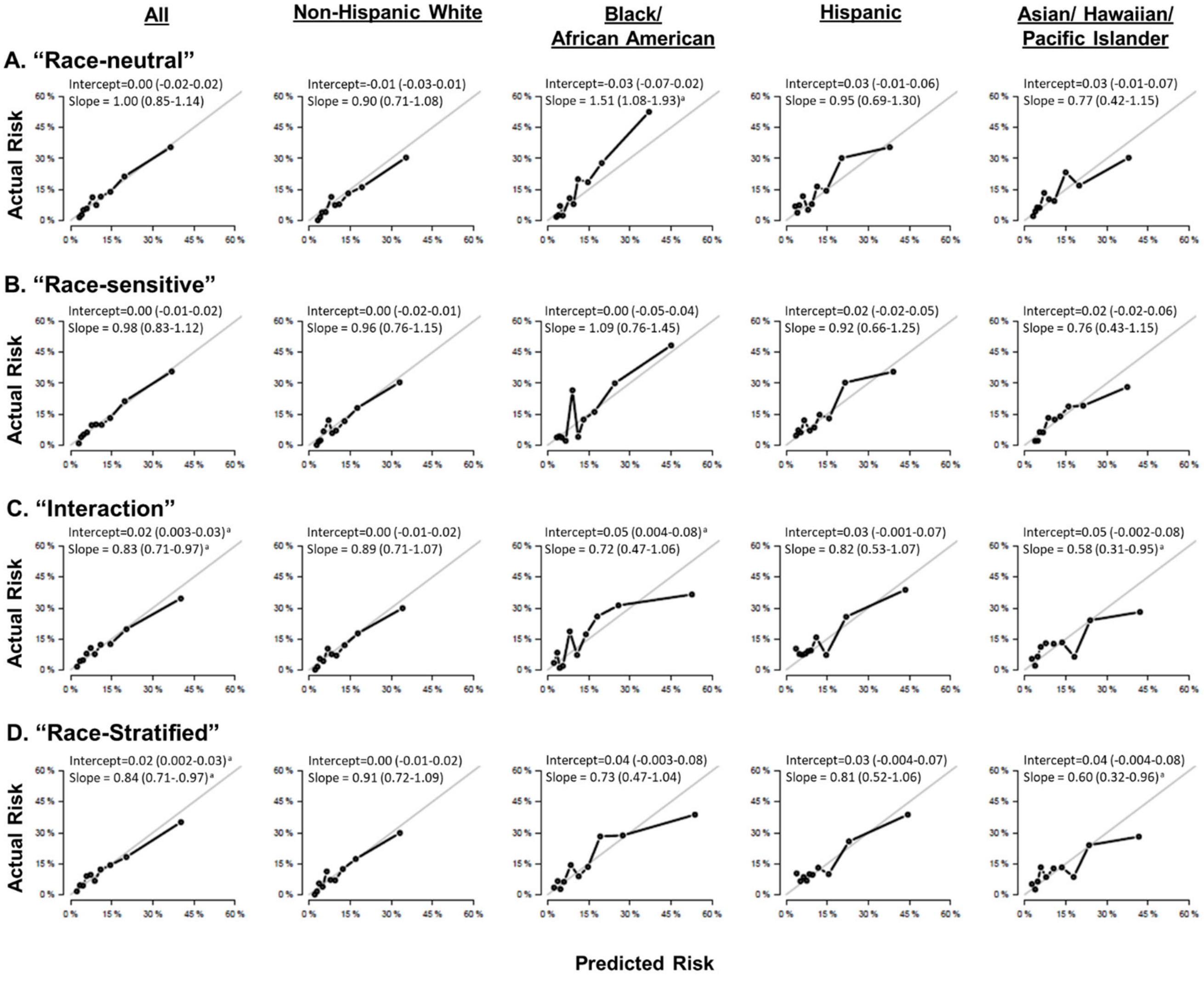

[a]Indicates that the 95%CI of the slope does not include 1; or the 95%CI of the intercept does not include 0. 95% CIs are obtained through bootstrapping.

© 2023 Khor S et al. *JAMA Network Open.*

eFigure 3. False-Positive Rates (FPR) and False-Negative Rates (FNR) at Different Risk Thresholds, by Model Type and Race and Ethnicity

The solid lines show FNR and the dashed lines show FPR.

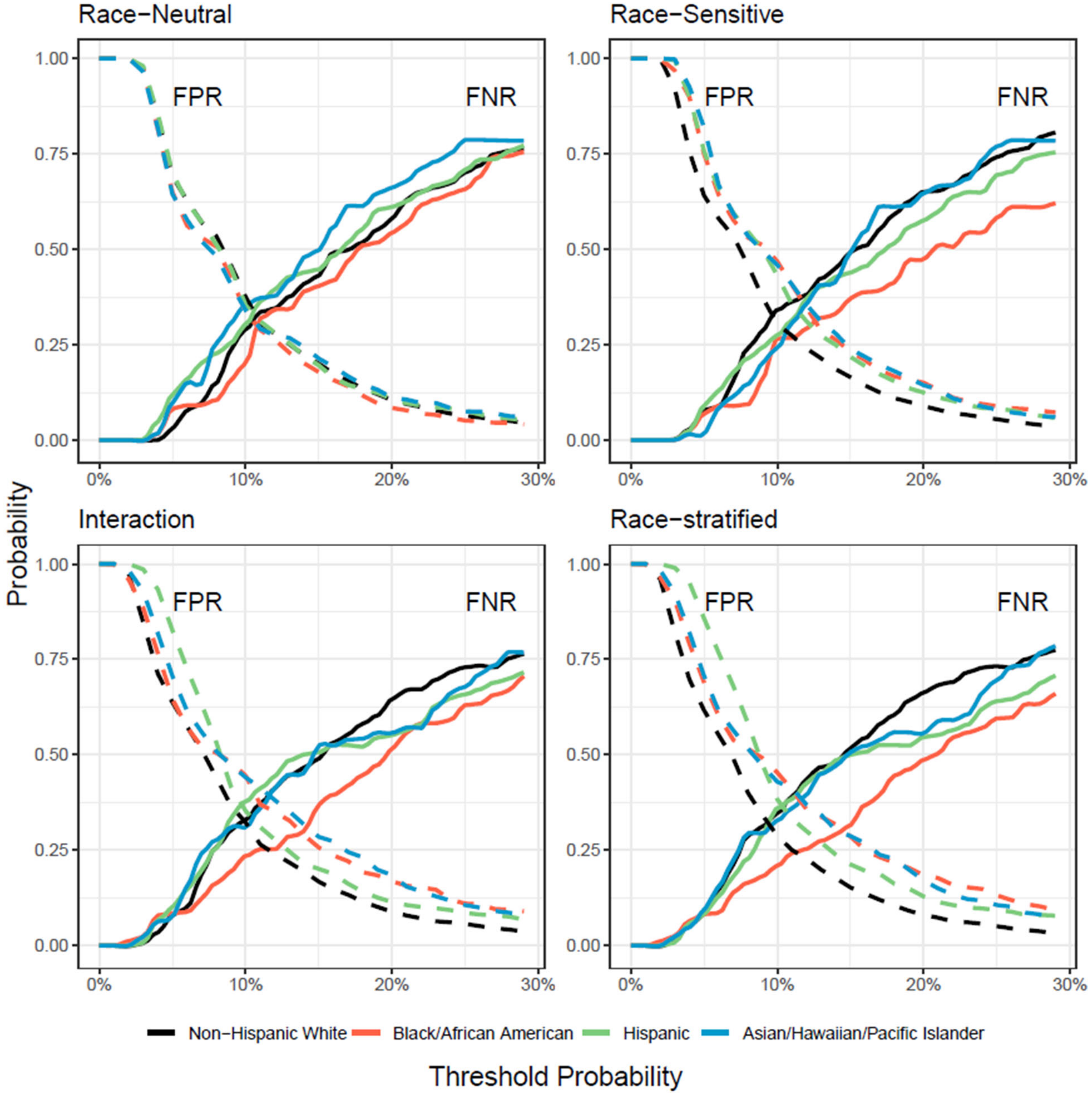

© 2023 Khor S et al. *JAMA Network Open.*

eFigure 4. Positive Predictive Value (PPV) and Negative Predictive Value (NPV) at Different Risk Thresholds, by Model Type and Race and Ethnicity

The solid lines show PPV and the dashed lines show NPV.

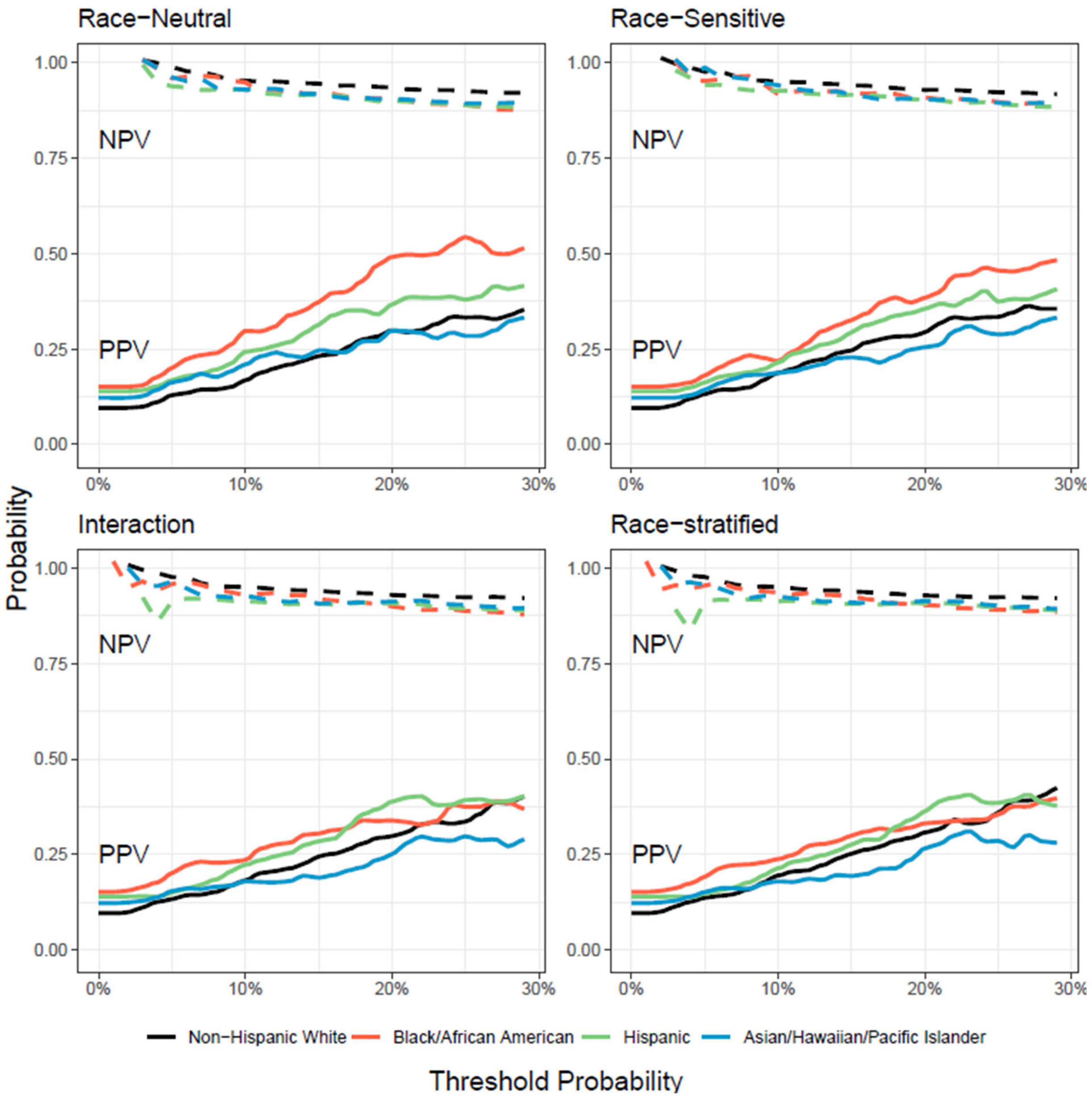

© 2023 Khor S et al. *JAMA Network Open.*

eReferences.

1. Hassett MJ, Uno H, Cronin AM, Carroll NM, Hornbrook MC, Ritzwoller D. Detecting Lung and Colorectal Cancer Recurrence Using Structured Clinical/Administrative Data to Enable Outcomes Research and Population Health Management. *Med Care*. 12 2017;55(12):e88-e98. doi:10.1097/MLR.0000000000000404
2. Hassett MJ, Ritzwoller DP, Taback N, et al. Validating Billing/Encounter Codes as Indicators of Lung, Colorectal, Breast, and Prostate Cancer Recurrence Using 2 Large Contemporary Cohorts. *Medical Care*. 2014;52(10):e65-e73. doi:10.1097/MLR.0b013e318277eb6f
3. Zafar SN, Hu CY, Snyder RA, et al. Predicting Risk of Recurrence After Colorectal Cancer Surgery in the United States: An Analysis of a Special Commission on Cancer National Study. *Ann Surg Oncol*. Aug 2020;27(8):2740-2749. doi:10.1245/s10434-020-08238-7
4. Derose SF, Contreras R, Coleman KJ, Koebnick C, Jacobsen SJ. Race and ethnicity data quality and imputation using U.S. Census data in an integrated health system: the Kaiser Permanente Southern California experience. *Med Care Res Rev*. Jun 2013;70(3):330-45. doi:10.1177/1077558712466293
5. Kleinberg J, Mullainathan S, Raghavan M. Inherent Trade-Offs in the Fair Determination of Risk Scores,. *arXiv*. 2016;doi:10.48550/ARXIV.1609.05807
6. Chouldechova A. Fair Prediction with Disparate Impact: A Study of Bias in Recidivism Prediction Instruments. *Big data*. 2017;5(2):153-163. doi:10.1089/big.2016.0047
7. Hedden B. On statistical criteria of algorithmic fairness. *Philosophy & Public Affairs*. 2021;49(2):209-231. doi:https://doi.org/10.1111/papa.12189
8. Van Calster B, McLernon DJ, van Smeden M, Wynants L, Steyerberg EW, initiative TGEdtapmotS. Calibration: the Achilles heel of predictive analytics. *BMC Med*. 12 16 2019;17(1):230. doi:10.1186/s12916-019-1466-7
9. Fong H, Kumar V, Mehrotra A, Vishnoi NK. Fairness for AUC via Feature Augmentation. 2021;
10. Hosmer DW, Lemeshow S. *Applied logistic regression*. Second edition. ed. Wiley series in probability and statistics Texts and references section. Wiley; 2000.
11. Hardt M, Price E, Srebro N. Equality of Opportunity in Supervised Learning. 2016;
12. Corbett-Davis S, Goel S. The measure and mismeasure of fairness: a critical review of fair machine learning. *arXiv*. 2018;(arXIv:1808.00023 [stat AP])

© 2023 Khor S et al. *JAMA Network Open.*
