# Supplementary material for: Racial and Ethnic Bias in Risk Prediction Models for Colorectal Cancer Recurrence When Race and Ethnicity Are Omitted as Predictors
Source: JAMA Netw Open. 2023 Jun 15;6(6):e2318495. doi: 10.1001/jamanetworkopen.2023.18495 (PMC10273018; doi:10.1001/jamanetworkopen.2023.18495)
Supplement: Supplement 2. — Data Sharing Statement [file jamanetwopen-e2318495-s002.pdf]

Data Sharing Statement

Khor. Racial and Ethnic Bias in Risk Prediction Models for Colorectal Cancer Recurrence When Race and Ethnicity Are Omitted as Predictors. *JAMA Netw Open*. Published June 15, 2023. doi:10.1001/jamanetworkopen.2023.18495

Data
Data available: No

Additional Information
Explanation for why data not available: The deidentified dataset used in this study can be made available on a reasonable request basis with institutional approvals.
